# Young age and return to play increase the likelihood of subsequent ACL reconstruction in football players: Data from the Swedish National Knee Ligament Registry

**DOI:** 10.1002/ksa.12580

**Published:** 2025-01-08

**Authors:** Anne Fältström, Magnus Forssblad, Alexander Sandon

**Affiliations:** ^1^ Region Jönköping County, Rehabilitation Centre Ryhov County Hospital Jönköping Sweden; ^2^ Unit of Physiotherapy, Department of Health, Medicine and Caring Sciences Linköping University Linköping Sweden; ^3^ Department of Molecular Medicine and Surgery, Stockholm Sports Trauma Research Center Karolinska Institute Stockholm Sweden

**Keywords:** ACL reconstruction, football, re‐injury, revision, soccer

## Abstract

**Purpose:**

To compare football players who have undergone one anterior cruciate ligament (ACL) reconstruction (ACLR) with those who have undergone a subsequent ACLR (revision or contralateral) regarding (1) demographics, (2) football‐related factors and (3) injury‐specific data.

**Methods:**

Players who voluntarily completed a football‐specific questionnaire available at the Swedish National Knee Ligament Registry website between April 2017 and September 2020 at the time of their primary ACL injury were included in the study. The questionnaire covered demographics, football‐related activities and injury‐specific factors. Subsequent ACLR registrations within 4 years of the primary ACLR were identified in December 2023. Data on game participation post‐primary ACLR were retrieved from the Swedish Football Association's administrative system in September 2022.

**Results:**

A total of 992 football players (66% men) were included, of whom 99 (10%) were registered for subsequent ACLRs. Univariable analysis showed that the following factors significantly increased the odds of a subsequent ACLR: female sex, younger age, a lower weight and body mass index, fewer years played, use of knee control exercises during warm‐up, more likely to plan a return to football, more game participation registered following the primary ACLR, and shorter time between injury and ACLR. Multivariable logistic regression analysis indicated that the odds of undergoing subsequent ACLR decreased significantly with each additional year of age (odds ratio [OR], 0.87; 95% confidence interval [CI], 0.83‒0.92, *p* < 0.01). Players using knee control exercises during warm‐up (OR, 1.71; 95% CI, 1.08‒2.72, *p* = 0.02), planning to return to football (OR, 2.74; 95% CI, 1.27‒5.91, *p* = 0.01), and participating in games after primary ACLR (OR, 1.81; 95% CI, 1.13‒2.91, *p* = 0.01) increased the odds of undergoing a subsequent ACLR.

**Conclusions:**

Younger age and returning to play after an ACLR significantly increase the likelihood of undergoing a subsequent ACLR in football players.

**Level of Evidence:**

Level IV.

**Abbreviations**
ACL: anterior cruciate ligament
ACLR: anterior cruciate ligament reconstruction
BMI: body mass index
CI: confidence interval
CT: computerised tomography
FOGIS: Swedish Football Association's Administrative IT System
IQR: interquartile range
MRI: magnetic resonance imaging
OR: odds ratio
SD: standard deviation
SNKLR: Swedish National Knee Ligament Registry

## INTRODUCTION

In Sweden, more than 90% of all anterior cruciate ligament (ACL) reconstructions (ACLRs) are recorded in the Swedish National Knee Ligament Registry (SNKLR) [6]. Approximately 40% of these ACLRs are performed on patients who sustained their injuries while playing football [6].

Returning to football after ACLR carries a high risk of re‐injury to the ipsilateral‐ or contralateral knee [10]. Such re‐injuries in football may be influenced by various factors, some of which might be preventable through targeted interventions. Since 2017, the SNKLR has included a specific questionnaire for football players, to capture football‐related factors [23]. These factors can be categorised into internal (demographics), external (football‐related) and injury‐specific variables, which are often used in models to explain the causes of sports injuries. However, it remains unclear whether these factors differ between individuals who undergo only a primary ACLR and those who require additional ACLR procedures. Understanding these differences could help identify factors that can be targeted for football‐specific injury prevention and provide deeper insights into the risk factors associated with subsequent ACLRs in football players. This study aimed to compare football players who have undergone one ACLR with those who have undergone a subsequent ACLR (revision or contralateral) regarding (1) demographics, (2) football‐related factors and (3) injury‐specific data within 4 years of follow‐up. The hypothesis was that females, younger age, small graft size, and players at elite level returning to football would be more likely to require a subsequent ACLR, while players using knee control exercises during warm‐up will be less likely to experience another ACLR.

## MATERIALS AND METHODS

### Participants

This registry study included those patients registered in the SNKLR [6] between April 2017 and September 2020 who completed a questionnaire addressing football‐related factors associated with their ACL injury. Inclusion criteria were completion of the questionnaire within 1 year before or up to 90 days after the registered primary ACLR, age ≤40 years, injury sustained while playing football at any level, absence of major concurrent knee injuries (such as fractures, posterior cruciate ligament injuries and surgically treated collateral ligament injury), nerve/vessel damage, and the use of either bone‐patellar‐tendon bone, semitendinosus/semitendinosus‐gracilis or quadriceps tendon autografts. Other grafts and allografts were excluded. Eligible patients in the SNKLR were matched to responses from the football‐specific questionnaire.

### Data collection

The questionnaire was originally developed in Germany in 2015 to investigate variables specific to football players, including demographics, football exposure, training, warm‐up practices, regeneration and decision‐making regarding return to play [17]. Its purpose was to enhance injury prevention strategies by providing prospective detailed knowledge about ACL injury characteristics specific to football players in different playing levels [17, 25, 26]. The questionnaire was later translated into Swedish, adapted for use in the SNKLR, and made available online in 2017 [23].

The questionnaire addressed three main categories: demographic, football‐related and injury‐specific data. The demographic data included sex, age, height, weight and body mass index (BMI). Football‐related data covered years played, level of play, playing position, preferred kicking leg, use of knee control exercises during warm‐up (answered as ‘Do you warm up using specific knee control exercises? [yes/no]’), and whether the player planned to return to football. Playing levels were categorised based on the Swedish football league system: elite level (top two Swedish divisions), high (division 1‒2), middle (men's division 3‒4, women's division 3), low (men's division 5‒8, women's division 4‒5) and recreational level [14].

Injury‐specific data included the injury mechanism (contact or non‐contact), whether the player started the game, pitch surface (grass, artificial grass, indoor and other), game time (minute) of injury, changes in club/division/coach before the season, use of new football shoes in the last month, shoe type at the time of injury, games played in the preceding 7 days and symptoms or injuries in the last 6 weeks. Post‐injury data included time to ACL diagnosis and whether diagnostic imaging (e.g., magnetic resonance imaging [MRI], radiography, computerised tomography [CT] and ultrasonography) was performed.

Additional data from SNKLR surgical records were collected, including injury dates, and surgery details (injured side, graft, ACLR date, surgically treated concomitant injuries and ACLR registrations). Subsequent ACLR data (ipsilateral or contralateral) were extracted up to 31 December 2023. To ensure comparable risk exposure, only subsequent ACLRs occurring within 4 years of the primary ACLR were included in the analysis.

Game participation after the primary ACLR was obtained from the Swedish Football Association's Administrative IT System (FOGIS), where all football players aged >12 years in Sweden must be registered to participate in games.

The study was approved by the Swedish Ethical Review Authority (Dnr: 2020‐03747 and 2021‐07044‐01) and by the SNKLR Board.

### Statistical analysis

The statistical analyses were performed using IBM SPSS Statistics for Windows (version 27.0; IBM Corp; Armonk, NY). Means ± standard deviation (SD) or medians and interquartile range (IQR) were calculated for descriptive statistics depending on the data level and normality. Between‐group comparisons were conducted between those who underwent one ACLR and those who underwent subsequent ACLRs using Student's *t*‐test for ratio scale data with normal distributions, Mann–Whitney *U*‐test for ordinal data or non‐normal distributions and chi‐squared test for nominal data as appropriate. Cohen's *d* values were reported for ratio scale data with the following effect size limits: 0.2, small effect; 0.5, medium effect; 0.8, large effect. The between‐group comparisons were conducted to select relevant variables for an univariable logistic regression analysis. Variables with *p* < 0.1 in the between‐group comparisons were used in the univariable logistic regression analysis to examine the association between demographics data, football‐related data, associated injuries and treatment data with the likelihood of undergoing a subsequent ACLR. In the final multivariable logistic regression analyses, variables with a significance level of *p* < 0.1 in the univariable logistic regression analysis were selected and checked for multicollinearity using a variance inflation factor > 5 assessed via the linear regression method. Weight was excluded due to multicollinearity. Eight variables (sex, age at the ACLR, BMI, years played, use of knee control exercises during warm‐up, plan to return to football, a registered game played after primary ACLR, and time between injury and ACLR) were then included in a multivariable logistic regression analysis using a backward procedure starting with the inclusion of all eight variables and then performing stepwise deletion of the variables with the highest *p* values. The results of the final step of the multivariable logistic regression analysis included the odds ratio (OR), confidence interval (CI) and *p* values. The level of significance was set at *p* < 0.05.

## RESULTS

From 1025 responses to the football‐specific questionnaire, 33 duplicates were excluded, leaving 992 players (650 men [66%] and 342 women [34%]) for analysis. The mean age was 24.4 ± 6.1 years for men and 20.9 ± 5.8 years for women (*p* < 0.01). Ninety‐nine (10%) of the 992 players underwent a subsequent (60 revision [61%] and 39 contralateral [39%]) ACLR, occurring an average of 2.3 ± 0.9 years after the primary ACLR. Follow‐up times exceeded 3.5 years for 94% of the players in one ACLR group and 93% of the subsequent ACLR group (*p* = 0.83).

### Demographics and football‐related factors

Univariate analysis indicated that subsequent ACLR was significantly associated with female sex, younger age, lower body weight and BMI, fewer years played, more frequent use of knee control exercises during warm‐up, more likely to plan to return to football, and higher game participation after primary ACLR (Table 1).

**Table 1:** Demographics and football‐related factors for players with one ACLR compared with players with subsequent ACLRs.

	Total cohort (*n* = 992)	One ACLR (*n* = 893)	Subsequent ACLR (*n* = 99)	*p*	Cohen's *d*	OR (95% CI; *p*)
Sex, *n* (%)				**0.01**		**1.75 (1.15‒2.66; <0.01)**
Female	342 (34)	296 (87)	46 (13)			
Male (%) (reference)	650 (66)	597 (92)	53 (8)			
Age at primary ACLR (years), mean (SD)	23.2 (6.3)	23.6 (6.3)	19.2 (4.5)	**<0.01**	**0.72**	**0.86 (0.82–0.90; <0.01)**
Age at primary ACLR, *n* (%)				**<0.01**		
11‒15 years	83 (8)	63 (76)	20 (24)			**14.50 (4.16‒50.58; <0.01)**
16‒20 years	313 (32)	265 (85)	48 (15)			**8.27 (2.53‒27.04; <0.01)**
21‒25 years	252 (25)	229 (91)	23 (9)			**4.59 (1.35‒15.56; 0.02)**
26‒30 years	204 (21)	199 (98)	5 (2)			1.15 (0.27‒4.88; 0.85)
31‒40 years (reference)	140 (14)	137 (98)	3 (2)			
Height (cm), mean (SD)	176.0 (8.9)^a^	176.1 (8.9)^a^	174.8 (9.3)	0.17		
Weight (kg), mean (SD)	74.7 (12.9)^b^	75.1 (12.9)^b^	71.4 (13.2)	**<0.01**	**0.28**	**0.98 (0.96‒0.99; <0.01)**
Body mass index (kg/m^2^), mean (SD)	24.0 (3.0)^a^	24.1 (2.9)^a^	23.2 (3.1)	**<0.01**	**0.29**	**0.90 (0.83‒0.97; <0.01)**
**Football‐related data**						
Years played, mean (SD)	14.8 (6.1)^c^	15.1 (6.2)^c^	12.3 (4.2)	**<0.01**	**0.46**	**0.92 (0.88‒0.96; <0.01)**
Level of play, *n* (%)				**0.02**		
Elite (top 2 Swedish divisions)	59 (6)	49 (83)	10 (17)			2.18 (0.82‒5.83; 0.12)
High (divisions 1–2)	197 (20)	167 (85)	30 (15)			1.75 (0.80‒3.81; 0.16)
Middle (divisions 3–4, men; 3, women)	243 (24)	217 (89)	26 (11)			0.92 (0.40‒2.09, 0.83)
Low (divisions 5–8, men; 4–5, women)	344 (35)	320 (93)	24 (7)			0.68 (0.30‒1.53, 0.35)
Recreational (reference)	149 (15)	140 (94)	9 (6)			
Playing position, *n* (%)				0.44		
Goalkeeper	37 (4)	32 (86)	5 (14)			
Defender	343 (35)	313 (91)	30 (9)			
Midfielder	389 (39)	244 (88)	45 (12)			
Forward	223 (22)	204 (91)	19 (9)			
Preferred kicking leg, *n* (%)				0.79		
Right	806 (81)	723 (90)	83 (10)			
Left	106 (11)	97 (92)	9 (8)			
Two‐footed	80 (8)	73 (91)	7 (9)			
Used knee control exercises during warm‐up, *n* (%)				**<0.01**		
Yes	212 (21)	176 (83)	36 (17)			**2.33 (1.50‒3.62; <0.01)**
No (reference)	780 (79)	717 (92)	63 (8)			
Planning a return to football, *n* (%)				**<0.01**		
Yes	740 (75)	649 (88)	91 (12)			**4.28 (2.05–8.94; <0.01)**
No (reference)	252 (25)	244 (97)	8 (3)			
Registered game played after primary ACLR, *n* (%)				**<0.01**		**1.38 (0.85–2.25; <0.01)**
Yes	499 (50)	430 (86)	69 (14)			
No (reference)	493 (50)	463 (94)	30 (6)			

*Note*: *p* Values in bold type are significant. Cohen's *d* values with the following effect size limits: 0.2, small effect; 0.5, medium effect; 0.8, large effect.

Abbreviations: ACLR, anterior cruciate ligament reconstruction; CI, confidence interval; OR, odds ratio; SD, standard deviation.

^a^ Four missing values.

^b^ One missing value.

^c^ Two missing values.

### Injury‐specific data

No significant differences were observed in injury‐specific variables between groups (Tables 2 and 3). However, players with subsequent ACLR had a shorter time between primary injury and primary ACLR compared with those with only one ACLR (Table 4).

**Table 2:** Data on injury occurrence for players with one ACLR compared with players with subsequent ACLRs.

	Total cohort (*n* = 992)	One ACLR (*n* = 893)	Subsequent ACLR (*n* = 99)	*p*
Injury mechanism, *n* (%)				0.52
Contact	380 (38)	339 (89)	41 (11)	
Non‐contact	612 (62)	554 (91)	58 (9)	
Started the game, yes, *n* (%)^a^	556 (84)	496 (89)	60 (11)	0.73
Pitch surface, *n* (%)				0.10
Grass	357 (36)	325 (91)	32 (9)	
Artificial grass, *n* (%)	525 (53)	463 (88)	62 (12)	
Indoor	97 (10)	92 (95)	5 (5)	
Other	13 (1)	13 (100)	0 (0)	
Game minute, median (IQR)^b^	35 (44)	35 (44)	30 (48)	0.70
Game minute, *n* (%)^b^				0.68
0–15	146 (24)	129 (88)	17 (12)	
16–30	140 (23)	122 (87)	18 (13)	
31–45	96 (16)	87 (91)	9 (9)	
46–60	71 (12)	64 (90)	7 (10)	
61–75	82 (14)	76 (93)	6 (7)	
76–90+	74 (12)	63 (85)	11 (15)	

Abbreviations: ACLR, anterior cruciate ligament reconstruction; IQR, interquartile range.

^a^ 663 (67%) players were injured in game.

^b^ 609 answers.

**Table 3:** Preparation and influencing factors for players with one ACLR compared with players with subsequent ACLRs.

	Total cohort (*n* = 992)	One ACLR (*n* = 893)	Subsequent ACLR (*n* = 99)	*p*
Club change for this season, *n* (%)				0.88
Yes	133 (13)	119 (89)	14 (11)	
No	859 (87)	774 (90)	85 (10)	
Division change this season, *n* (%)				0.36
No	715 (72)	649 (91)	66 (9)	
Yes, to a higher division	197 (20)	172 (87)	25 (13)	
Yes, to a lower division	80 (8)	72 (90)	8 (10)	
Coach change, *n* (%)				0.69
No	668 (67)	605 (91)	63 (9)	
During the season	210 (21)	186 (89)	24 (11)	
Before the season	114 (12)	102 (89)	12 (11)	
New football shoes last month, *n* (%)				0.78
Yes	168 (17)	150 (89)	18 (11)	
No	824 (83)	743 (90)	81 (10)	
Shoe type at time of injury, *n* (%)				0.17
Firm ground, permanent studs	613 (62)	555 (91)	58 (9)	
Soft ground, screw‐in studs	18 (2)	15 (83)	3 (17)	
Artificial grass	236 (24)	205 (87)	31 (13)	
No studs	57 (6)	54 (95)	3 (5)	
Other	68 (7)	64 (94)	4 (6)	
Games played in the previous 7 days, *n* (%)	a	b	c	0.74
0	336 (36)	305 (91)	31 (9)	
1	443 (47)	395 (89)	48 (11)	
2 or more	161 (17)	146 (91)	15 (9)	
Symptoms or injury in the last 6 weeks before the ACL injury, yes, *n* (%)	218 (22)	199 (91)	19 (9)	0.53
Distribution of symptoms and injuries per body part, *n* (%)				
Head	5 (1)	5 (100)	0 (0)	
Neck	5 (1)	4 (80)	1 (20)	
Back	41 (4)	40 (98)	1 (2)	
Hip	24 (2)	21 (88)	3 (2)	
Groin	31 (3)	30 (97)	1 (3)	
Thigh	43 (4)	39 (91)	4 (9)	
Knee	135 (14)	124 (92)	11 (8)	
Ankle/foot	56 (6)	53 (95)	3 (5)	

Abbreviations: ACL, anterior cruciate ligament; ACLR, anterior cruciate ligament reconstruction; IQR, interquartile range.

^a^ Fifty‐two missing values.

^b^ Forty‐seven missing values.

^c^ Five missing values.

**Table 4:** Time until ACL diagnosis, imaging and data from the Swedish National Knee Ligament Registry for players with one ACLR compared with players with subsequent ACLRs.

	Total cohort (*n* = 992)	One ACLR (*n* = 893)	Subsequent ACLR (*n* = 99)	*p*	OR (95% CI; *p*)
Days until ACL diagnosis, median (IQR)^a^	22 (53)^a^	24 (58)^a^	18 (46)	**0.04**	0.99 (0.99‒1.00; 0.11)
Days until ACL diagnosis, *n* (%)				0.50	
1–7 days	261 (26)	229 (88)	32 (12)		
8–14 days	136 (14)	122 (90)	14 (10)		
15–31 days	215 (22)	196 (91)	19 (9)		
32–365 days	333 (34)	301 (90)	32 (10)		
>365 days	44 (4)	42 (95)	2 (5)		
Imaging, yes, *n* (%)	742 (75)	665 (89)	77 (11)	0.47	
Magnetic resonance imaging	737 (74)	662 (90)	75 (10)		
Radiography	581 (59)	523 (90)	58 (10)		
Computerised tomography	19 (2)	19 (100)	0 (0)		
Ultrasonography	49 (5)	42 (86)	7 (14)		
Time between injury and ACLR (months), median (IQR)	6.3 (8.9)^b^	6.5 (9.4)^b^	4.6 (4.7)	**<0.01**	**0.98 (0.96‒1.00; 0.05)**
Time from ACLR and answering the survey (days), median (IQR)	15 (55)	15 (55)	18 (54)	0.67	
Injured knee, *n* (%)				0.77	
Right	545 (55)	492 (90)	53 (10)		
Left	447 (45)	401 (90)	46 (10)		
Graft, autografts, *n* (%)				0.55	
Semitendinosus	888 (90)	802 (90)	86 (10)		
Bone‐patellar‐bone	71 (7)	63 (89)	8 (11)		
Quadriceps	33 (3)	28 (85)	5 (15)		
Graft diameter (mm), mean (SD)	8.7 (0.8)^c^	8.7 (0.8)^c^	8.7 (0.8)	0.90	
Graft diameter, *n* (%)^c^				0.06	
<8 mm	103 (10)	86 (83)	17 (17)		1.97 (0.69‒5.63; 0.21)
8–9.5 mm	771 (78)	701 (91)	70 (9)		1.41 (0.59‒3.35; 0.21)
>10 mm (reference)	116 (12)	104 (90)	12 (10)		
Concomitant injuries treated at primary ACLR, *n* (%)					
Cartilage	62 (6)	59 (95)	3 (5)	0.21	
Medial meniscus	199 (20)	184 (92)	15 (8)	0.20	
Medial meniscus suture	116 (12)	109 (94)	7 (6)	0.13	
Lateral meniscus	335 (34)	305 (91)	30 (9)	0.44	
Lateral meniscus suture	101 (10)	91 (90)	10 (10)	0.98	

*Note*: *p* Values in bold type are significant.

Abbreviations: ACL, anterior cruciate ligament; ACLR, anterior cruciate ligament reconstruction; CI, confidence interval; IQR, interquartile range; OR, odds ratio; SD, standard deviation.

^a^ Three missing values.

^b^ Ten missing values.

^c^ Two missing values.

### Multivariable logistic regression analysis

Multivariable logistic regression analysis showed that the odds of undergoing a subsequent ACLR decreased significantly with each additional year of age. Conversely, the use of knee control exercises during warm‐up, planning to return to football and participating in games after the primary ACLR were associated with increased odds of subsequent ACLR (Table 5).

**Table 5:** Statistically significant factors of undergoing a subsequent ACLR in the final step of the multivariable backwards logistic regression analysis (*n* = 992; 893 with primary ACLR and 99 with subsequent ACLRs).

	OR (95% CI)	*p*
Age at primary ACLR	0.87 (0.83‒0.92)	**<0.01**
Used knee control exercises during warm‐up		
Yes	1.71 (1.08‒2.72)	**0.02**
No (reference)		
Plan a return to football		
Yes	2.74 (1.27‒5.91)	**0.01**
No (reference)		
Registered for game play after primary ACLR		
Yes	1.81 (1.13‒2.91)	**0.01**
No (reference)		

*Note*: *p* values in bold type are significant.

Abbreviations: ACLR, anterior cruciate ligament reconstruction; CI, confidence interval; OR, odds ratio.

## DISCUSSION

The most important findings of the present study were that younger age, planning to return to football and actual return to football were associated with subsequent ACLR. Additionally, players who reported using knee control exercises during warm‐up were also more likely to undergo a subsequent ACLR. However, factors such as playing position, preferred kicking leg, injury occurrence details, preparation and influencing factors for ACL injuries, and surgery data (e.g., graft type, graft size and concomitant injury) did not differ between players with a primary and subsequent ACLR. While these results partially confirmed our hypotheses, the unexpected association between knee control exercises and subsequent ACLR warrants careful interpretation.

The multivariable logistic regression model showed that younger age, use of knee control exercises during warm‐up, and plans or actual return to football were significant independent factors of subsequent ACLR. Each additional year of age decreased the odds of subsequent ACLR by 13%. Players aged 11‒15 years had 14.5 times higher odds of subsequent ACLR, and those aged 16‒25 years had approximately four to eight higher odds compared to players aged 31‒40 years. This is in line with previous studies reporting that young age is associated with subsequent ACLR or ACL injury [4, 5, 18, 22, 28] and the odds of re‐rupture or revision surgery decreased by 32%‒53% for each 1‐year increase in age [4, 22]. However, the elevated risk in younger athletes may reflect greater football exposure and activity levels [22], as well as mismatches between sport return timelines and functional readiness. For instance, returning to high‐demand sports within the first post‐operative year has been associated with a sixfold increased risk of subsequent ACL injury [12].

Return to football, particularly to Level I sport, has been previously linked to higher odds of subsequent ACL injury or ACLR [4, 5, 12, 28]. Regarding activity level and football exposure, players who plan to return to football and players who were registered for game participation in FOGIS after their primary ACLR had between one and four times increased odds of undergoing a subsequent ACLR. Two of the three players who stated that they planned to return had game registration after primary ACLR [14]. Thus, planning to return does not necessarily equate to actual return as some players may retire from the sport, re‐injure before game registration, change sports, or play at a recreational level.

The finding that using knee control exercises during warm‐up was associated with higher odds of subsequent ACLR is intriguing. While such exercises are a recommended intervention for primary ACL injury prevention [2], there is limited evidence on their effectiveness in preventing secondary ACL injuries [11]. In the current study, only one fifth of all players stated that they used knee control exercises during warm‐up at the primary ACL injury. It is unlikely that these exercises independently increase the risk of subsequent ACLR. A plausible explanation is that players practising knee control exercises may have higher exposure to football, thereby increasing their risk of re‐injury. Additionally, we lack detailed information on how players interpreted and performed these exercises, including their type, quality, duration and frequency. This limitation underscores the need to interpret these results cautiously.

The univariable logistic regression analysis identified female sex, lower weight and BMI and fewer years of football experience as factors increasing the odds of subsequent ACLR. However, these factors were excluded in the final multivariable model. Previous studies report conflicting findings regarding sex differences, with some showing higher risks for contralateral ACLR in females [5], a lower risk for re‐ruptures or revision [4], or no sex differences [21]. In football players, females have been reported to experience a higher incidence of secondary ACL injury compared with males (27% vs. 10%) [13].

The role of BMI in subsequent ACLR is also debated. While some studies report no association between BMI and revision ACLR [4], others have found that a BMI <25 kg/m^2^ triples the odds of contralateral rupture or ACLR [5]. Moreover, risk profiles incorporating factors such as hamstring strength, quadriceps‐to‐hamstrings ratio, psychological readiness and return to sport have included BMI as a contributing variable in some contexts but not others [1, 9].

Regarding football experience, we found that fewer years of playing football increased the odds of subsequent ACLR, potentially reflecting an age‐related effect. In elite athletes, shorter career durations have been associated with higher preseason ACL injury rates [20]. Conversely, longer experience in younger players may increase exposure to high‐level competition and training intensity, raising the risk of ACL injuries [27].

In surgery data, only the time between the injury and ACLR differed significantly between groups, with shorter times observed in those with subsequent ACLR. Early ACLR (<3–6 months) has been associated with higher activity levels and greater risks of revision surgery [3, 8, 15, 24] or contralateral ACLR [8]. This association can be explained by the fact that early ACLR is usually recommended for active young patients who want to return to pivoting sports [15]. Female football players who returned to football had a shorter time between ACL injury and ACLR than those who did not return [7]. Those with early ACLR (<3‒6 months from injury) had a higher activity level 1 year post‐operatively than those with ACLR > 6 months from injury [15] and were younger; it is speculated that patients with delayed ACLR may be more mentally and physically attuned to living with an injured knee [3, 15, 24].

Finally, factors such as playing position, preferred kicking leg, injury mechanism, pitch surface and preparation details (e.g., shoe type, recent gameplay and changes in club/division/coach) showed no significant differences between groups.

### Strengths and limitations

One strength of the present study is the homogeneous cohort regarding age, sport and grafts used. However, its generalisability may be limited. The football‐specific questionnaire included previously unexplored factors that might influence the likelihood of subsequent ACLR. The questionnaire has been used in the SNKLR since 2017, allowing for at least 3 years of follow‐up for subsequent ACL injury registration and 2 years for game participation registration in FOGIS after primary ACLR. Subsequent ACLR and game registrations could have occurred after these years. While subsequent ACLR and game registrations may have occurred beyond this period, the analysis was limited to subsequent ACLRs within 4 years to ensure comparable risk exposure. Re‐injuries not reconstructed and ACLRs not registered in SNKLR were not included. Data from FOGIS provided insights into post‐ACLR game participation, but the length of players' football careers remains unknown. The mean career length of football players following ACLR is approximately between 4 and 5 years [19]. The voluntary nature of the football‐specific questionnaire introduces potential response bias, and the response rate is unknown. Many players were excluded due to either completing the questionnaire more than 90 days post‐ACLR or having ACLR dates more than 365 days prior, a strategy employed to reduce recall bias and ensure responses were relevant to the primary ACLR. In Sweden, the average time between injury and surgery has ranged from 400 to 500 days [6] meaning patients could complete the questionnaire long before ACLR. There are indications to perform lateral extra‐articular tenodesis (LET) augmentation in patients aged <25 years and for patients wishing to return to high‐risk sports, but most commonly indicated in revision ACLR and pivot shift Grade ≥2 [16]. Since LET data have only been recorded in SNKLR since June 2018, and only six cases were registered in this cohort, its role remains unclear. Finally, the football‐specific questionnaire has not been validated for reliability or accuracy, which is a notable limitation.

## CONCLUSION

Younger age and returning to play after an ACLR significantly increase the likelihood of undergoing subsequent ACLR in football players. These findings underscore the importance of tailored rehabilitation strategies and informed decision‐making regarding return to sport to mitigate re‐injury risks.

## AUTHOR CONTRIBUTIONS

All authors designed the study, read and approved the final manuscript and were involved in data interpretation. Anne Fältström analysed the data and wrote the manuscript.

## CONFLICT OF INTEREST STATEMENT

The authors declare no conflicts of interest.

## ETHICS STATEMENT

The study was approved by the Swedish Ethical Review Authority (Dnr: 2020‐03747 and 2021‐07044‐01) and followed the principles of the Declaration of Helsinki.

## Data Availability

De‐identified data can be provided on reasonable request.
